# Supplementary material for: Active site specificity profiling datasets of matrix metalloproteinases (MMPs) 1, 2, 3, 7, 8, 9, 12, 13 and 14
Source: Data Brief. 2016 Feb 22;7:299–310. doi: 10.1016/j.dib.2016.02.036 (PMC4777984; doi:10.1016/j.dib.2016.02.036)
Supplement: Supplementary file 10 — Supplementary material [file mmc10.zip › WebPICS_hMMP3_T_1%/P1prime.html]

 

PICS results


|  |  |
| --- | --- |
| **P1prime\_C**  10 in 199 sites   5.0 %    effects > 10 perc. pnts.  (vice-versa in brackets)  P3\_I: 21.5 (12.6) |  |
| **P1prime\_F**  8 in 199 sites   4.0 %    effects > 10 perc. pnts.  (vice-versa in brackets)  P2\_W: 10.5 (21.0)   P1\_D: 26.9 (10.3)   P3prime\_T: 18.0 (10.3) |  |
| **P1prime\_I**  30 in 199 sites   15.1 %    effects > 10 perc. pnts.  (vice-versa in brackets)  P3\_A: 14.6 (18.2) |  |
| **P1prime\_M**  6 in 199 sites   3.0 %    effects > 10 perc. pnts.  (vice-versa in brackets)  P2\_Q: 25.8 (10.3)   P2prime\_H: 13.7 (13.7)   P3prime\_T: 26.3 (11.3) |  |
| **P1prime\_Q**  11 in 199 sites   5.5 %    effects > 10 perc. pnts.  (vice-versa in brackets)  P1\_G: 18.3 (11.2)   P2prime\_Q: 30.9 (30.9)   P3prime\_H: 15.2 (27.8)   P3prime\_Q: 21.3 (19.5) |  |
| **P1prime\_V**  31 in 199 sites   15.6 %    effects > 10 perc. pnts.  (vice-versa in brackets)  P1\_F: 12.1 (46.9)   P1\_P: 10.4 (17.7)   P2prime\_I: 11.5 (16.2)   P3prime\_G: 10.7 (11.1) |  |
| **P1prime\_W**  6 in 199 sites   3.0 %    effects > 10 perc. pnts.  (vice-versa in brackets)  P2\_S: 26.3 (11.3)   P1\_G: 57.7 (19.2)   P2prime\_M: 13.2 (11.3)   P3prime\_H: 13.7 (13.7) |  |
